# Chinese college students collaborative mobile-assisted language learning experience and flow as a key factor for further adoption

**DOI:** 10.3389/fpsyg.2023.1165332

**Published:** 2023-09-13

**Authors:** Ling Hu, Dan Hei, Hui Wang, Xuanrui Dai

**Affiliations:** ^1^School of Foreign Languages, Hunan University, Changsha, China; ^2^Henan Institute of Economics and Trade, Zhengzhou, China

**Keywords:** collaborative MALL experience, flow theory, behavioral intention, collaboration and share, mobile technology adoption, MCLL model

## Abstract

**Introduction:**

In recent years, the widespread shift toward online learning in higher education has led to a notable increase in the utilization of collaborative mobile-assisted language learning (MALL). However, the efficacy and implementation of MALL in college settings remain subjects of ongoing scholarly debate. To gain deeper insights into the experiences of Chinese college students with collaborative MALL and investigate factors that may influence their intentions for further adoption, this study proposed a comprehensive model that integrates the updated Unified Theory of Acceptance and Use of Technology (UTAUT) and flow theory.

**Methods:**

The model aimed to assess the relationship between flow and various antecedents, including perceived cost, social influences, perceived mobility, collaboration, and knowledge sharing, which shape students’ intentions to adopt collaborative MALL. A survey was conducted among a sample of 831 students from 32 provinces and autonomous regions.

**Results:**

The data analysis revealed that while 73% of participants reported having experienced collaborative MALL, overall adoption levels among Chinese college students are still in its initiative stage of adoption. Furthermore, variations were observed in the experiences of students from different majors and level of education. Importantly, the assessment of the proposed Mobile Collaborative Language Learning (MCLL) Model demonstrated the significant role of flow in predicting the adoption of collaborative MALL among Chinese college students.

**Discussion:**

The study concludes with suggestions for future research opportunities based on the research findings, aiming to enhance our understanding and application of collaborative MALL in higher education contexts.

## 1. Introduction

Mobile learning has played a vital role in preserving human interaction during the pandemic (Godwin-Jones, 2011; Burston, 2013; Adedoyin and Soykan, 2020; Ali et al., 2021; Zhang and Zhu, 2021), as over 90% of students worldwide have suffered from school closures (Zhou et al., 2020; Kamal et al., 2021; UNESCO, 2021) during the COVID-19 outbreak. A large number of higher education courses were completed online *via* mobile technology. As a result, online collaboration has become a more frequent form of learning both in class and outside of class, serving as both a course requirement and a natural aspect of learning in human society (Hodges et al., 2020; Yilmaz and Kostur, 2021).

Various categories of online collaborative activities (Zhou, 2016; Zhang, 2018; Chen et al., 2022; Fu, 2022; Gao and Zhao, 2022; Huang, 2022; Li, 2022; Zhang and Zhu, 2022; Dong, 2023) were identified and reported. However, research on MALL in college settings remains a topic of debate as both positive and negative outcomes of collaborative MALL have been reported. Recent research emphasizes the importance of understanding students’ perceptions and acceptance of Mobile-Assisted Language Learning (MALL) for its effective integration in education (Cheon et al., 2012; Abu-Al-Aish and Love, 2013). As online collaboration gains significant traction in language learning, it becomes imperative to gain fresh insights and perspectives directly from students.

Moreover, the past three years have witnessed a substantial shift toward online language courses, encompassing a diverse range of collaborative learning activities conducted exclusively through digital platforms. Practitioners have directed their attention to critical questions, such as “How do students experience collaborative MALL?” and “What factors influence their experiences and willingness to further embrace this learning approach?” However, limited empirical research exists on the variations in college students’ intentions for collaborative learning and their perceptions of the factors related to flow, as well as their willingness to adopt technology in the future. This may be partly due to the fact that MALL was initially regarded as an individualized and customized approach to learning, and research on online collaboration has only recently received significant attention. Furthermore, early research on MALL primarily focused on innovative features and functions (Sharples, 2000; Terras and Ramsay, 2012), behavioral changes (Hubbard, 2009; Kukulska-Hulme, 2009; Godwin-Jones, 2011), supportive environments (Laurillard and Pachler, 2007; Lan et al., 2013; Sun et al., 2020) and effectiveness (Thornton and Houser, 2005; Stockwell, 2010, 2013; Kukulska-Hulme, 2012), while little has been done to explore users’ psychological and mental changes.

Hence, the purpose of this study is to explore the experiences of Chinese college students with collaborative MALL and uncover the factors that may influence their intentions to further adopt this approach. The study contributes to the existing academic literature by offering valuable insights into the experiences and intentions of college students in China regarding collaborative MALL. The findings of this study are expected to inform educational practitioners, policymakers, and researchers in developing effective strategies for integrating collaborative MALL into language learning contexts, thereby enhancing the overall learning experiences and outcomes for students.

## 2. Literature review

### 2.1. Collaborative learning

For over half a century, educators and researchers have emphasized the importance of collaborative learning (Hwang and Fu, 2019), which has been widely utilized as an effective instructional method in traditional learning environments (Dillenbourg and Schneider, 1995; Watanabe and Swain, 2007). Collaborative learning is defined as a method in which two or more learners work together in pairs or groups to achieve shared goals (Barkley et al., 2014). Unlike individual learning, collaborative learning allows learners to share their resources and skills, such as asking for information, evaluating, and monitoring each other’s ideas (Dillenbourg, 1999).

Moreover, collaborative learning facilitates students’ reflection on their past experiences and thoughts (Hwang et al., 2011), leading to better social interaction when members actively engage with one another (Leung and Chiu, 2008). The benefits of collaborative learning for language learning over individual learning have been supported by numerous studies, including those conducted in face-to-face settings (e.g., Pressley et al., 1995; Slavin, 1996; Johnson and Johnson, 1999).

### 2.2. Collaborative learning and MALL

In recent years, MALL has gained increasing prominence in the field of language learning, providing rich learning opportunities for learners (Laurillard and Pachler, 2007; Virvou et al., 2012; Lan et al., 2013; Burston, 2015; Abe et al., 2017; Shao et al., 2020; Sun et al., 2020; Burston and Arispe, 2022) and characterized by its convenience, connectivity, personalization, and interaction (Sharples, 2000; Terras and Ramsay, 2012). The use of varied technologies recently to support collaborative learning has gained increasing attention due to technological advances (Liu et al., 2016; UNESCO, 2021; Burston and Arispe, 2022) and the nature of learning (as discussed by Vygotsky, 1962; Dillenbourg, 1999; Kock, 2007) since around 2008 (Hwang and Fu, 2019).

Researchers have increasingly recognized “collaboration” as the core of mobile-assisted language learning (MALL), rather than just an element of the learning process. For example, Liu et al. (2009) investigated the development of collaborative learning activities to enhance students’ learning in mobile learning environments. Koh and Hill (2009) differentiated various online collaborative activities, from participating in discussion boards to engaging in small group activities. Hwang et al. (2016) highlighted that product-oriented collaboration may lead to more frequent practice. Andujar (2016) emphasized the discourse created through interaction and collaboration in the process of constructing meaning. Other studies (Ogunduyile, 2013; Ilic, 2015) have also reported collaboration resulting from the adoption of multiple technologies and media. A number of studies investigate how collaboration facilitates students’ learning of vocabulary (Sun et al., 2020; Al-Ahdal and Alharbi, 2021), reading (Lan et al., 2013; Lin, 2014; Hazaea and Alzubi, 2016), writing (Andujar, 2016; Teng, 2022), and speaking (Kirsch, 2016), as well as the application of certain mobile devices or technologies (Andres and Shipps, 2010; Head, 2014; Reinhardt and Ross, 2019).

Although the initial purposes of collaborative MALL studies may vary, researchers have identified various categories of online collaborative activities (Zhang, 2018; Chen et al., 2022; Fu, 2022; Gao and Zhao, 2022; Huang, 2022; Li, 2022; Zhang and Zhu, 2022; Dong, 2023). It is interesting to see the diverse range of collaborative activities in MALL that have been identified and categorized by researchers. These activities seem to have different goals and objectives, and they vary in terms of the level of teacher facilitation, the duration of the task, and the type of final outcome. It is also noteworthy that some of these activities are more formal and structured, such as the course-required grouping task, while others are more informal and spontaneous, such as the always-with-you collaboration. It seems that collaborative MALL can provide learners with flexible and diverse learning opportunities that can cater to different learning needs and preferences.

Positive researchers have suggested that collaborative MALL can enhance language learning and offer convenient access to information anytime and anywhere (Kukulska-Hulme and Viberg, 2018; Hwang and Fu, 2019; Hsu and Lin, 2021). Besides allowing learners to collaborate with peers or native speakers from different backgrounds and cultures, collaborative MALL also provide learners a more inclusive and diverse learning environment and engage them in shared learning experiences. Some of the benefits of collaborative MALL identified by Kukulska-Hulme and Viberg (2018) include improved motivation and engagement, increased opportunities for language practice and feedback, enhanced language learning strategies, better development of communicative competence, and improved social and intercultural awareness.

However, negative findings have also been reported. For instance, Hsu (2013) found that inappropriate awareness of the educational benefits and effectiveness of MALL may lead to reluctance and resistance toward the approach. Additionally, Luo and Chen (2019) reported that a majority of Chinese students sampled used mobile technology for language learning for less than 20 min at a time. Zhang and Pérez-Paredes (2019) demonstrated that students were not regularly and actively engaged with mobile English learning resources. Moreover, Nguyen and Takashi (2021) revealed that Vietnamese and Japanese learners rarely used mobile devices to study English outside the classroom, despite expressing a desire to do so. Ramli et al. (2010) found that learners in their study struggled with learning transfer because they did not perceive mobile phones as useful for learning.

Overall, positive findings suggest that collaborative MALL can facilitate learning and provide convenient access to information anytime and anywhere, while negative findings suggest that inappropriate awareness and limited usage of mobile technology can result in reluctance and resistance to this learning approach. Therefore, it is crucial to consider factors that play important roles in the effective adoption of collaborative MALL in language learning contexts.

### 2.3. Factors related to collaborative MALL

The effectiveness of collaborative MALL as a language learning approach may depend on various factors, including linguistic, behavioral, psychological, technical and social factors, as well as students’ perceptions, attitudes, and motivation. Technical factors refer to the quality and accessibility of the mobile devices and networks, as well as the compatibility of the collaboration tools with different platforms and devices (Brahmasrene and Lee, 2012; Chen and Wu, 2016). Social factors include the social norms, communication styles, and cultural backgrounds of the learners, as well as the quality of the relationships between the learners and the teacher or facilitator (Kukulska-Hulme and Shield, 2008; Chen and Wu, 2016; Chen et al., 2018; Cho and Castaneda, 2019). All these factors may affect the effective implement of collaborative MALL.

Over years, many studies have investigated various factors related to students’ willingness to use technology for learning. The UTAUT, proposed by Venkatesh and Davis (2000), Venkatesh et al. (2003), has been widely applied in numerous studies (Briz-Ponce et al., 2016; Sumak and Sorgo, 2016; Ai et al., 2018; Yakubu and Dasuki, 2019; Bower et al., 2020; Xu and Zhang, 2020) to explore factors that influencing users behavior change intention. The UTAUT proposes four key constructs that influence the intention and subsequent adoption of various technologies, namely: performance expectancy, effort expectancy, social influence, and enabling conditions. The concept of technology acceptance or adoption implies that technologies are innovations that pose a challenge to users. Flow theory, on the other hand, emphasizes the symbiotic relationship between challenges and the skills needed to meet those challenges. According to this theory, one’s skills must meet a given challenge before they can reach a state of flow (Csikszentmihalyi, 1990; Nakamura and Csikszentmihalyi, 2002; Abuhamdeh, 2020). As students seek to master new challenges, such as collaborative MALL, they develop greater levels of skill. In the process of mastering these new skills, they must progressively identify increasingly complex challenges to create an ideal match for their skills. Flow, therefore, invokes a growth principle in which a more complex set of capacities is sought after and developed (Admiraal et al., 2011).

In recent years, flow has been considered an important factor that can influence or mediate students’ behavior in accepting new technologies. Flow theory has been viewed a new perspective in education for measuring students’ learning experience and outcomes (Marks, 2000; Kiili, 2005; Appleton et al., 2006; Bressler and Bodzin, 2013). According to Csikszentmihalyi (1990), flow is an optimal state of focused concentration in which distractions are minimized, and the individual enjoys an autonomous interaction with the activity, functioning at their fullest capacity (Whalen and Henker, 1999). Flow theory emphasizes the relationship between challenges and the skills needed to meet them, where an individual’s skills must meet the challenge to achieve a state of flow (Csikszentmihalyi, 1990; Nakamura and Csikszentmihalyi, 2002; Abuhamdeh, 2020). Flow has also been introduced to the field of MALL, where it is regarded as an important factor impacting or mediating students’ academic outcomes. As students seek to master new challenges like collaborative MALL, they develop greater levels of skill, identifying increasingly complex challenges to create an ideal match for their skills. Flow invokes a growth principle, where a more complex set of capacities is sought and developed (Admiraal et al., 2011; Franciosi, 2011; Guo et al., 2016; Xu and Zhang, 2020).

### 2.4. The proposed mobile collaborative language learning model

Drawing on the literature review and the specific context of our study, the Mobile Collaborative Language Learning Model (MCLL) is proposed. The model identifies four motivational factors that are likely to influence students’ flow experience in collaborative MALL. These factors include Perceived Cost, Social Influences, Perceived Mobility, and Collaboration and Share. These factors are hypothesized to have impacts on students’ flow experience, and that flow, in turn, will influence their intention for further adoption of collaborative MALL.

**Figure 1:**
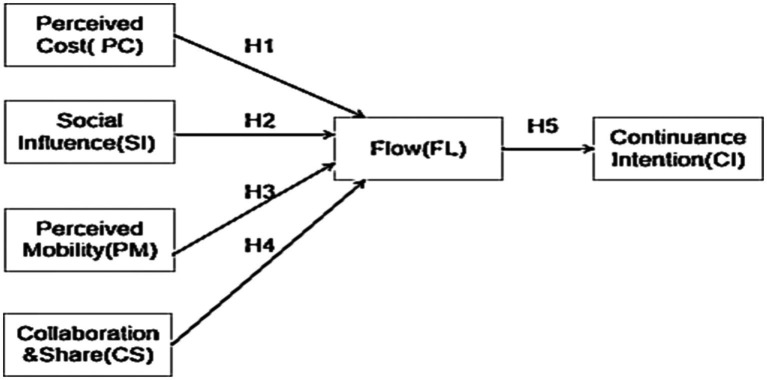
The proposed model: the MCLL model.

Based on the proposed model, five hypothesis have been raised to describe the relationships between the factors shown in Figure 1.

Perceived cost refers to the cost, time and energy that users perceive when using products or receiving services. In the context of MALL environment, perceived cost is defined as various expenses, time and energy paid by users when users learn collaboratively in a mobile context. Generally, high perceived costs will reduce users’ flow and diminish their intention for further adoption. Therefore, the first hypothesis is proposed as follow:

*H1*: Perceived Cost will have positive effect on Flow.

Technical factors refer to the mobility and accessibility of the mobile devices and networks, as well as the compatibility of the collaboration tools with different platforms and devices (Brahmasrene and Lee, 2012; Chen and Wu, 2016). Perceived Mobility allows users to obtain various information and services through mobile devices anytime, anywhere. The mobility of mobile device leads to efficiency and practicality, which are two main advantages of mobile learning. Timely response will influence learners’ flow in the collaborative MALL experience. Therefore, we hypothesize:

*H2*: Perceived Mobility will have positive effect on Flow.

In educational contexts, collaboration refers to two or more team members engaging in planning or problem-solving by continuous and interdependent interactions (Kock, 2007; Kukulska-Hulme and Viberg, 2018; Hwang and Fu, 2019; Hsu and Lin, 2021). We assume collaboration and share will be a very critical factor of affecting students’ flow experience and ultimately influence the behavioral intention of collaborative MALL Therefore, we hypothesize:

*H3*: Collaboration and Share will have positive effect on Flow.

Social factors include the social norms, communication styles, and cultural backgrounds of the learners, as well as the quality of the relationships between the learners and the teacher or facilitator (Kukulska-Hulme and Shield, 2008; Chen and Wu, 2016; Chen et al., 2018; Cho and Castaneda, 2019). Based on the literature, social influences can play a determinant influence on individual’s behavioral intention (Taylor and Todd, 1995; Hoffman and Novak, 1996; Hsu and Lu, 2004; Dağhan and Akkoyunlu, 2016). The adoption of collaborative MALL will be influenced by peers’ recommendation and teachers’ encouragement. Therefore, we propose that it will influence learners’ flow in the collaborative MALL experience.

*H4*: Social Influences will have positive effect on Flow.

Literature reveals a positive relationship between an experience of flow and learning in general (Skinner et al., 1990; Marks, 2000; Kiili, 2005) and learning in second language acquisition (Egbert, 2003; Franciosi, 2011; Kiili et al., 2012; Delforge et al., 2019). Based on the literature, we assume students are facing a challenging innovation and the state of flow can be an evident predictor to measure students’ intention for further collaborative MALL adoption. Therefore, the fifth hypothesis is proposed as follow:

*H5*: Flow will have a positive effect on Continuance Intention for further collaborative MALL experience.

## 3. Research design

The purpose of this study is to provide a comprehensive description of college students’ experiences in China regarding collaborative Mobile-Assisted Language Learning (MALL). Thus, the first research question was raised.

(1) What are the status quo of Collaborative MALL experience of Chinese college students?

Additionally, the study aims to examine the significance of the concept of flow in understanding students’ intentions to further adopt collaborative MALL and to explore the influence of the factors on students’ behavior and how they impact the experience of flow, hence, the MCLL model have been proposed and five hypotheses need to be verified (See 2.5 for details). Specifically, two research questions are proposed.

(2) How do Perceived Cost, Social Influences, Perceived Mobility and Collaboration and Share relate to the flow of Chinese college students’ collaborative MALL?
(3) How does flow relate to Chinese college students’ intention for further adoption of collaborative MALL?

Of the five hypotheses, hypothesis 1, 2, 3, and 4 are to answer research question 2. While hypothesis 5 is to answer research question 3.

### 3.1. Research method and instruments

Quantitative survey has been adopted for the study. Data were collected through a questionnaire called “The Questionnaire of College Students’ Collaborative MALL Experience in China.” “College students” in this study are students enrolled in higher educational institutes in China, including junior colleges, colleges, and universities.

The questionnaire used in this study is composed of four sections. The first section is focused on gathering personal information such as the students’ age group, gender, and grade. The second section aims to identify whether students have prior experience with Collaborative MALL. It consists of only one item: “Have you ever experienced any collaborative MALL?” Participants who answered “no” would have the questionnaire process terminated, while only those who had collaborative MALL experience would continue. The third section focuses on students’ specific experience of Collaborative MALL, for example, “how long have you experienced collaborative MALL?.” The last section is a 15-item scale specifically developed for this study, which aims to investigate the relationship between several factors and students’ collaborative MALL experience, with reference to two previous studies (Bower et al., 2020; Xu and Zhang, 2020). For example, “collaborative MALL enables me to learn more knowledge.” Responses were measured on a 5-point Likert scale ranging from “strongly disagree” to “strongly agree.” The development of the scale is based on the two integrating theories, namely the Unified Theory of Acceptance and Use of Technology (UTAUT) and flow theory, aiming to explore the psychological mechanisms underlying students’ adoption of collaborative MALL. An initiative pilot study has been conducted among 47 students from three universities in China. Items with ambiguity were revised and a second pilot test was then conducted among another group of 63 students from the same three universities. Data analysis based on 51 participants with collaborative MALL experience showed a high value for reliability coefficient that guaranteed the implementation of the main study (Table 1). Data collected in both the pilot studies and main study were online, using the same service provider – Wenjuanxing.

**Table 1:** Reliability test of the scale items.

***N* of Items**	***n***	**Cronbach α**
15	51	0.911

### 3.2. Sampling and data collection

Using a random sampling process, the questionnaire survey was conducted through an online questionnaire platform, namely Wenjuanxing (Questionnaire Star) between 13th and 20th in December 2022. To reach the target population, varied methods have been adopted including QQ groups, WeChat groups and a services particularly provided by the Wenjuanxing. At the end of the data collection period, a total of 831 respondents were recruited from 32 provinces and autonomous regions, which were representative to a certain extent. Among the respondents, 607 were college students who had experience with collaborative MALL. Respondents who had no experience with collaborative MALL were excluded for the analysis. The sample overview is presented in Table 2.

**Table 2:** Overview of the respondents.

**Province**	**Total**	**Collaborative MALL experience**			
		**Experienced**	**Percentage**	**Not experienced**	**Percent**
Jiangsu	145	129	15.5	16	1.9
Hunan	91	63	7.6	28	3.4
Zhejiang	73	67	8.1	6	0.7
Guangdong	71	31	3.7	40	4.8
Fujian	64	57	6.9	7	0.8
Liaoning	58	50	6.0	8	1.0
Anhui	48	27	3.2	21	2.5
Henan	39	22	2.6	17	2.0
Shandong	35	30	3.6	5	0.6
Jiangxi	25	11	1.3	14	1.7
Guangxi	23	13	1.6	10	1.2
Sichuan	17	13	1.6	4	0.5
Hebei	16	10	1.2	6	0.7
Hubei	16	12	1.4	4	0.5
Ningxia	15	10	1.2	5	0.6
Shaanxi	12	8	1.0	4	0.5
Chongqing	11	9	1.1	2	0.2
Heilongjiang	9	3	0.4	6	0.7
Yunnan	8	6	0.7	2	0.2
Gansu	7	3	0.4	4	0.5
Jilin	8	5	0.6	3	0.4
Beijing	7	4	0.5	3	0.4
Shanxi	7	5	0.6	2	0.2
Hainan	5	4	0.5	1	0.1
Shanghai	5	3	0.4	2	0.2
Mongolia	4	4	0.5	0	0.0
Xinjiang	3	2	0.2	1	0.1
Guizhou	2	1	0.1	1	0.1
Tianjin	3	2	0.2	1	0.1
Qinghai	2	2	0.2	0	0.0
Tibet	1	0	0.0	1	0.1
Macau	1	1	0.1	0	0.0
	831	607	73	224	27

### 3.3. Data analysis procedures

To answer research question 1, data was subjected to both descriptive and inferential analysis using SPSS 26.0. Descriptive analysis allowed for a comprehensive examination of the data, while inferential analysis enabled the exploration of potential relationships and associations among variables.

For research questions 2 and 3, a structural equation model (SEM) was employed to examine the underlying relationships and factors influencing students’ intention to further adopt collaborative Mobile-Assisted Language Learning (MALL). Specifically, the software programs AMOS 24.0 and SPSS 26.0 were utilized to conduct the analysis. The selection of AMOS was based on its capability to simultaneously model measurement paths, capturing the relationships between latent variables and their observed indicators, and structural paths, representing the theoretical relationships among latent variables (Chin, 2010).

The assessment of the measurement model’s goodness-of-fit was achieved through two widely recognized absolute fit measures: the likelihood-ratio chi-square statistic (χ2) and the root mean square error of approximation (RMSEA) (Hair et al., 1998; Kline, 2005; Kim and Garrison, 2009). These measures provided valuable insights into the overall fit of the model.

Furthermore, the validity of the model was examined using the Kaiser-Meyer-Olkin (KMO) value and Bartlett’s Test through factor analysis, ensuring the robustness and appropriateness of the measurement model (Ramayah and Suki, 2006). To evaluate the reliability of the constructs, Cronbach’s coefficient alpha was employed, as it is a widely accepted measure of internal consistency reliability (Nunnally and Bernstein, 1994; Hair et al., 1998). This analysis provided valuable insights into the consistency and stability of the constructs being measured.

Subsequently, the structural model was tested, examining the hypothesized relationships among the five variables. This involved examining R-square values and path coefficients to determine the extent to which the proposed factors influenced students’ intention to further adopt collaborative MALL (Hur, 2007; Watanabe and Swain, 2007; Xu and Zhang, 2020; Zhang and Zhu, 2022). By assessing the magnitude and statistical significance of the path coefficients and R-square values, insights were gained into the factors that significantly impacted students’ behavior and their intention to adopt collaborative MALL. Specifically, this analysis aimed to ascertain the significance of flow in understanding students’ intention to further adopt collaborative MALL, and the ways in which flow was influenced by various factors that shaped students’ behavior.

## 4. Results

Three questions have been proposed in this study. The first is to describe Chinese college students collaborative MALL experiences and the next two are to examine the influential factor – flow for students’ intention for further adoption of collaborative MALL based on the five hypotheses.

### 4.1. Chinese college students collaborative MALL experiences

#### 4.1.1. Collaborative MALL as a mainstream but new learning experience for Chinese college students

Table 3 shows that out of the total 831 respondents, 73% (607) reported having experience with collaborative MALL. This indicates that collaborative MALL is a relatively mainstream learning approach among Chinese college students, and suggests that these students are becoming increasingly comfortable with constructing and sharing knowledge through collaborative MALL, both inside and outside the classroom. However, the effectiveness of collaborative MALL still needs to be further investigated.

**Table 3:** Overview of students collaborative MALL experience.

**Items**	**Categories**	**Total**	**Percent**
Collaborative MALL experience	With collaborative MALL experience	607	73
	Without collaborative MALL experience	224	27
Gender	Male	269	44.3
	Female	338	55.7
Age group	18-22	534	88.0
	23-27	58	9.6
	28-31	12	2.0
	Over 32	3	0.5
Majors	Science and engineering	171	28.3
	Liberal arts	335	55.2
	Arts	101	16.6
Education level	Junior college	108	17.8
	Undergraduate	420	69.2
	Postgraduate	79	13.0

Among the 607 respondents with collaborative MALL experience, 59% were female, which is slightly higher than the male respondents. Junior college students and undergraduates make up the majority, accounting for 87% of the sample. The age range of the respondents is between 18 and 22, which is typical for Chinese college students.

It is worth noting that more undergraduate students report having collaborative MALL experience compared to junior college students. This difference may be attributed to the varying language learning outcomes required by the national curriculum. While language learning is compulsory in most higher education programs, the specific learning requirements differ across programs. Junior college students are generally required to achieve lower language outcomes compared to undergraduate students, which may limit their opportunities to experience collaborative MALL activities.

Students in liberal arts programs were found to have the highest frequency of collaborative MALL experience, while art students reported the least experience. It’s important to note that language majors are included in the liberal arts category, which may explain the higher frequency of collaborative MALL experience in this group. As language courses often require a significant amount of practice and communication, students in these programs may have more opportunities to engage in collaborative MALL and as a result, have a more intensive experience.

Table 4 presents the findings on Chinese college students’ collaborative MALL experience in terms of their behavior. The first aspect is the overall duration or history of their adoption. More than 60% of the students reported having less than a year of collaborative MALL experience, indicating that while the approach has been introduced, it is still a relatively recent experience and is at early stage compared to other MALL learning methods. Data analysis also shows that the frequency of students’ collaborative MALL experience is low. Specifically, 63% of respondents reported having collaborative MALL experience less than once a month or only once per semester, highlighting the relatively recent adoption of this approach by Chinese college students. In fact, the options for this item were revised based on feedback from pilot study respondents who reported limited online collaboration due to inefficiency and difficulty. As a result, many reported only experiencing collaborative MALL once or twice per semester. This low frequency may be due to the fact that many respondents are non-language majors and have only experienced collaborative MALL in their college foreign language courses, which make only a small portion of their overall credits. Additionally, collaborative MALL experiences, such as group discussions and presentations, are only a part of their course activities or assignments.

**Table 4:** Chinese college students’ collaborative MALL behaviour.

**Items**	**Categories**	**N**	**Percent (%)**	**Cumulative Percent (%)**
Duration of collaborative MALL experience	< 3 months	127	20.92	20.92
	> 3-6 months	172	28.34	49.26
	>6 -12 months	94	15.49	64.74
	> 1 -2 years	118	19.44	84.18
	> 2 years	96	15.82	100.00
Frequency of collaborative MALL experience in the last 3 months	1-2 times every 3 months	137	22.57	22.57
	1-2 times every 2 months	124	20.43	43.00
	1-2 times per month	123	20.26	63.26
	1-2 times per week	144	23.72	86.99
	More than 3 times per week	79	13.01	100.00
Duration of single collaborative MALL activity	< 30 minutes	194	31.96	31.96
	> 30 minutes-1 hour	208	34.27	66.23
	> 1 hour-2 hours	130	21.42	87.64
	> 2 hours	75	12.36	100.00
Most frequently adopted collaborative MALL activities	Discussion and exploration grouping	268	24.1	24.1
	Course required grouping task	269	24.1	48.2
	Always with you collaboration	313	28.1	76.3
	Online self-study grouping	264	23.7	100
**Total**		607	100.0	100

The reported duration of single collaborative MALL activities also indicates the relatively new experience of it among Chinese college students. Over 66% of students reported that their single collaborative MALL experience activity lasted less than one hour, while only 12% of them reported that their single collaborative MALL experience activity lasted more than two hours (Table 4).

The respondents were asked to select two activities that they use most frequently for collaborative MALL. The results showed a roughly balanced distribution of four commonly adopted collaborative MALL activities. Among the four activities, “Discussion and exploration grouping” and “Course required grouping task” tend to be more synchronous and are more commonly associated with course-related and teacher-initiated activities, while “Always with you collaboration” and “Online self-study grouping” are more asynchronous, less course-related, and more student-initiated (Gao and Zhao, 2022). Besides, it is interesting to note that among the four collaborative MALL activities, “Always with you collaboration” is the most frequently reported, and the two learner-initiated activities together occupy over 50% of the total share.

The analysis of students’ overall experience shows that collaborative MALL has been widely introduced and adopted by Chinese college students. However, the analysis of students’ specific behavior indicates that collaborative MALL is still relatively new. This suggests that students and teachers may still be in the process of exploring the claimed positive effects of collaborative MALL proposed by many research studies.

#### 4.1.2. Collaborative MALL experience differences due to educational level and major differences

As shown in Table 5, data analysis reveals no significant differences (*p > 0.05*) of learners from different education levels in their overall duration or history of collaborative MALL learning and their adoption of the two commonly applied collaborative activities, namely “discussion and exploration grouping” and “course required grouping task,” both of which are typically assigned by course teachers. These findings demonstrate the consistency and similarities of Chinese college students’ collaborative MALL experiences.

**Table 5:** Collaborative MALL experience differences due to level of education differences.

**Items**	**Categories**	**Education Levels N (%)**			**Total**	***χ2***	***p***
		**Junior college**	**Undergraduate**	**Postgraduate**			
Duration of collaborative MALL experience	< 3 months	24(22.22)	87(20.71)	16(20.25)	127(20.92)	9.526	0.300
	> 3-6 months	34(31.48)	115(27.38)	23(29.11)	172(28.34)		
	>6 -12 months	21(19.44)	60(14.29)	13(16.46)	94(15.49)		
	> 1 -2 years	20(18.52)	80(19.05)	18(22.78)	118(19.44)		
	> 2 years	9(8.33)	78(18.57)	9(11.39)	96(15.82)		
Frequency of collaborative MALL experience in the last 3 months	1-2 times every 3 months	21(19.44)	98(23.33)	18(22.78)	137(22.57)	19.827	0.011*
	1-2 times every 2 months	32(29.63)	67(15.95)	25(31.65)	124(20.43)		
	1-2 times per month	22(20.37)	86(20.48)	15(18.99)	123(20.26)		
	1-2 times per week	24(22.22)	107(25.48)	13(16.46)	144(23.72)		
	More than 3 times per week	9(8.33)	62(14.76)	8(10.13)	79(13.01)		
Duration of single collaborative MALL activity	< 30 minutes	25(23.15)	148(35.24)	21(26.58)	194(31.96)	22.463	0.001**
	> 30 minutes-1 hour	33(30.56)	151(35.95)	24(30.38)	208(34.27)		
	> 1 hour-2 hours	25(23.15)	82(19.52)	23(29.11)	130(21.42)		
	> 2 hours	25(23.15)	39(9.29)	11(13.92)	75(12.36)		
Most frequently adopted collaborative MALL activities	Discussion and exploration grouping	44(40.74)	183(43.57)	41(51.90)	268(44.15)	2.490	0.288
	Course required grouping task	58(53.70)	218(51.90)	37(46.84)	313(51.57)	0.925	0.630
	Always with you collaboration	77(71.30)	139(33.10)	48(60.76)	264(43.49)	62.029	0.000**
	Online self-study grouping	36(33.33)	206(49.05)	27(34.18)	269(44.32)	12.380	0.002**

*p < 0.05; **p < 0.01.

The analysis reveals significant differences among learners of different education levels in terms of the frequency of collaborative learning (χ^2^ = 19.827, *p* = 0.011 < 0.05). Postgraduates (31.65%) and junior college students (29.63%) are found to be more frequently engaged in collaborative MALL than the average level of 20.43%. However, the study also indicates that the overall adoption of collaborative MALL in China’s higher education is limited.

The differences among learners with different education levels in terms of the time duration of a single collaborative MALL learning activity are even more significant (*χ*^2^ = 22.463, *p* = 0.001 < 0.01). Postgraduates spent a longer time in a single collaborative MALL learning activity than junior college and undergraduate students. Since collaborative MALL requires interactions, sharing, and constructing new meaning through negotiations, the relatively short duration of the activity means that collaborative MALL has not yet fully functioned, and its effect has not been fully explored.

Despite demonstrating preferences for two informal collaborative MALL activities, learners with different education levels showed significant differences in their actual adoption of these activities. Junior college and postgraduate learners significantly preferred the “Always with you collaboration” (*χ*^2^ = 62.029, *p* = 0.000 < 0.01), whereas undergraduates showed a significant preference for “Online self-study grouping” (*χ*^2^ = 12.380, *p* = 0.002 < 0.01).

Table 6 displays the variations in collaborative MALL experience among learners based on their different majors. The results indicate that there are no significant differences in three items, including the duration of a single collaborative MALL activity and the two most frequently adopted collaborative MALL activities (*p* > 0.05). The learners from different majors behaved similarly in these three aspects. However, there are significant differences (*p* < 0.05) in four items, namely, the overall duration of collaborative MALL experience, the frequency of MALL experience in the past three months, and the other two most frequently adopted collaborative MALL activities.

**Table 6:** Collaborative MALL experience differences due to major differences.

**Items**	**Categories**	**Major N (%)**			**Total**	***χ*2**	***p***
		**Science and engineering**	**Liberal arts**	**Arts**			
Duration of collaborative MALL experience	< 3 months	47(27.49)	52(15.52)	28(27.72)	127(20.92)	38.588	0.000**
	> 3-6 months	62(36.26)	79(23.58)	31(30.69)	172(28.34)		
	> 6-12 months	23(13.45)	53(15.82)	18(17.82)	94(15.49)		
	> 1-2 years	23(13.45)	82(24.48)	13(12.87)	118(19.44)		
	> 2 years	16(9.36)	69(20.60)	11(10.89)	96(15.82)		
Frequency of collaborative MALL experience in the last 3 months	1-2 times every 3 months	43(25.15)	69(20.60)	25(24.75)	137(22.57)	19.651	0.012*
	1-2 times every 2 months	30(17.54)	62(18.51)	32(31.68)	124(20.43)		
	1-2 times per month	41(23.98)	64(19.10)	18(17.82)	123(20.26)		
	1-2 times per week	42(24.56)	85(25.37)	17(16.83)	144(23.72)		
	More than 3 times per week	15(8.77)	55(16.42)	9(8.91)	79(13.01)		
Duration of single collaborative MALL activity	< 30 minutes	61(35.67)	94(28.06)	39(38.61)	194(31.96)	10.571	0.103
	> 30 minutes-1 hour	59(34.50)	123(36.72)	26(25.74)	208(34.27)		
	> 1 hour-2 hours	31(18.13)	80(23.88)	19(18.81)	130(21.42)		
	> 2 hours	20(11.70)	38(11.34)	17(16.83)	75(12.36)		
Most frequently adopted collaborative MALL activities	Discussion and exploration grouping	61(35.67)	165(49.25)	42(41.58)	268(44.15)	8.792	0.012*
	Course required grouping task	90(52.63)	172(51.34)	51(50.50)	313(51.57)	0.131	0.937
	Always with you collaboration	81(47.37)	128(38.21)	55(54.46)	264(43.49)	9.790	0.007**
	Online self-study grouping	84(49.12)	145(43.28)	40(39.60)	269(44.32)	2.655	0.265

*p < 0.05 **p < 0.01.

The overall duration of collaborative MALL experience differed significantly (*χ*^2^ = 38.588, *p* = 0.000 < 0.01) among students from different majors. Liberal arts students reported a longer duration of collaborative MALL experience than students from other categories. Over 40% of liberal arts students had more than a year of overall collaborative MALL experience, compared to 22% of science and engineering students and 24% of arts students. This suggests that students’ collaborative MALL experience varies significantly based on their major, which indicates that the factors that influence students’ collaborative MALL experience may vary.

The frequency of collaborative MALL (*χ*^2^ = 19.651, *p* = 0.012 < 0.05) also showed significant differences. Liberal arts and science and engineering students reported higher frequency levels of collaborative MALL experience than arts students. Arts students reported a 31.68% frequency of once or twice a month collaborative MALL experience in their studies, which is significantly higher than that of the other two groups of students.

Among the four most frequently adopted collaborative MALL activities, two activities, “Course required grouping task” and “Online self-study grouping,” showed no significant differences among the three student groups. “Course required grouping task” is generally assigned by the course teacher, and thus, the even distribution is acceptable. However, the other two activities, “Discussion and exploration grouping” (*χ*^2^ = 8.792, *p* = 0.012 < 0.05) and “Always with you collaboration” (*χ*^2^ = 9.790, *p* = 0.007 < 0.01), showed significant differences. The analysis indicates that science and engineering and arts students had more opportunities to experience learner-initiated collaborative MALL learning, while liberal arts students were more teacher-initiated. This suggests that language teachers need to be more alert to technological advancements and be ready to integrate new collaborative MALL activities in their course planning and implementation.

### 4.2. Flow as a key factor to influence Chinese college students’ collaborative MALL adoption: test of the five hypotheses

To explore the influential factors of collaborative mobile-assisted language learning (MALL) experience among college students in China, a scale was developed, and five hypotheses were proposed. AMOS 24.0 and SPSS 26.0 were applied for the two stage analysis process: analysis of the measurement model and analysis of the structural model.

The measurement model was analyzed through item reliability examination using Cronbach’s coefficient alpha analysis. The results (Table 7) indicated that the reliability of the instrument was within acceptable levels (>0.7) as suggested by Hair et al. (1998), with Cronbach’s alpha estimates for constructs ranging between 0.711 (Perceived Cost) and 0.893 (Flow).

**Table 7:** Reliability and validity test of the questionnaire items.

**Construct**	**Items**	**Loading**	**Cronbach’s alpha**
PM (Perceived Mobility) Flow (Flow) PC (Perceived Cost) CS (Collaboration & Share) SI (Social Influence) CI (Continuance Intention)	PM1 PM2 FL1 FL2 FL3 PC1 PC2 CS1 CS2 CS3 SI2 SI3 CI1 CI2 CI3	0.772 0.802 0.680 0.696 0.742 0.722 0.804 0.650 0.647 0.664 0.740 0.768 0.783 0.748 0.673	0.813 0.893 0.711 0.877 0.774 0.891

The Kaiser-Meyer-Olkin (KMO) value (Table 8) was 0.960, and the Bartlett’s Test of the questionnaire was significant, indicating that the principal component factor analysis was acceptable (Ramayah and Suki, 2006). Hence, all the constructs in the proposed model demonstrated acceptable validity and reliability.

**Table 8:** KMO and Bartlett’s test of the questionnaire.

Method		Value
Kaiser-Meyer-Olkin Measure of Sampling Adequacy.		.960
Bartlett’s Test of Sphericity	Approx. Chi-Square	6732.159
	*df*	105
	Sig.	.000

The overall fit indices (Table 9) showed that the proposed model had a good fit to the data, which was the first step in analyzing the measurement model (Kim and Garrison, 2009), suggesting that the measurement model was acceptable.

**Table 9:** Summary of overall fit indices for the measurement model.

**Model**	**NFI**	**GFI**	**AGFI**	**CFI**	***X/df***	**RMSEA**	**RMR**
Measurement model	0.974	0.963	0.943	0.985	2.263	0.046	0.035
Recommend value	>0.9	>0.9	>0.9	>0.9	1< <3	<0.08	<0.05

**Figure 2:**
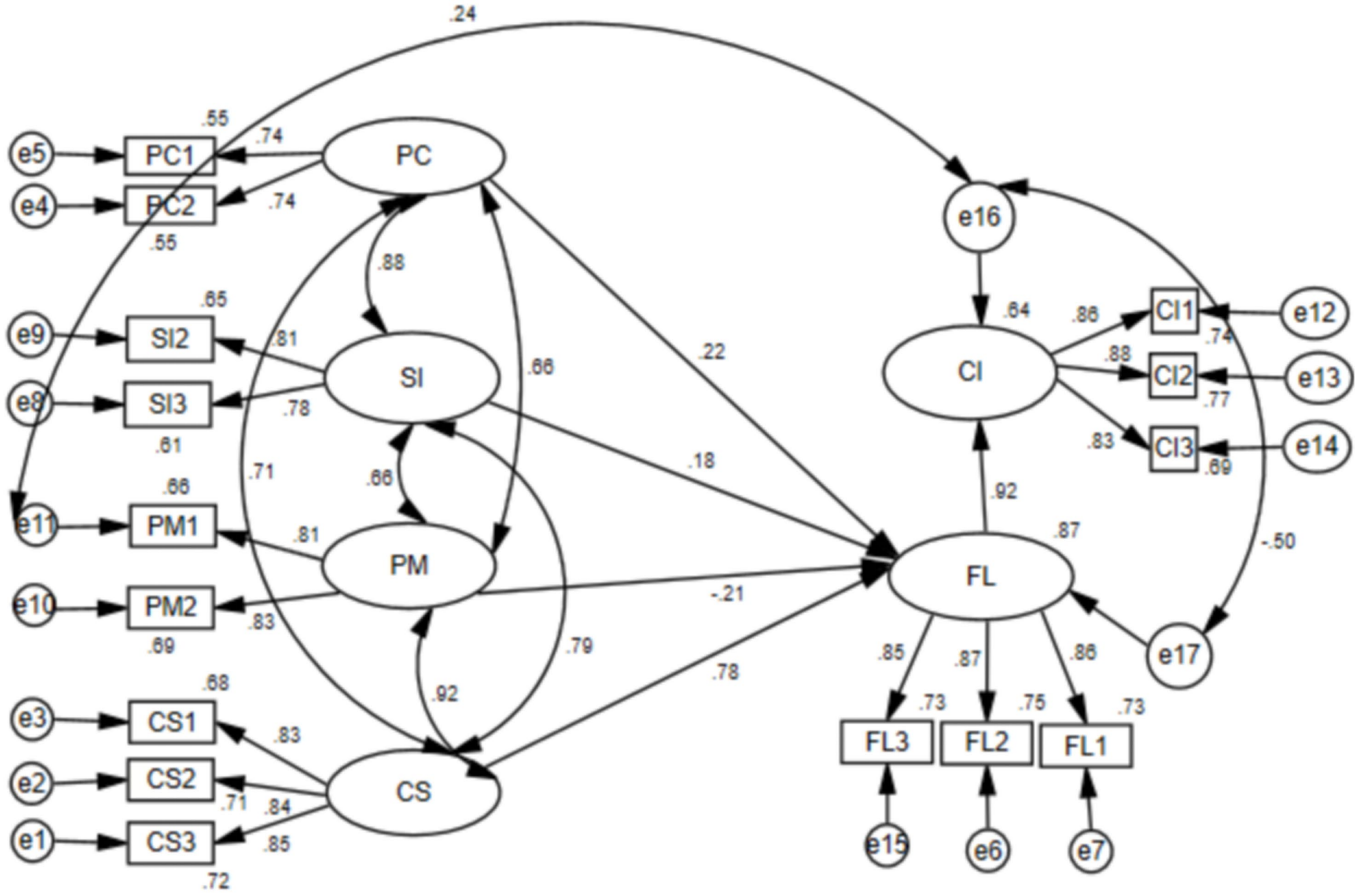
The MCLL model: flow as a key factor.

Following this, the structural model was evaluated in AMOS 24.0 to test the relationships between the constructs proposed in the collaborative MALL. The results of hypothesis testing and path coefficients with their respective significance levels are presented in Table 10 and Figure 2.

**Table 10:** Test of the five hypotheses.

**Hypothesis**	**Effects**	**S.E.**	**C.R.**	***p*-value**	**Path coefficients**	
H1	PC→FL	.119	2.018	.044*	.224	Support
H2	SI→FL	.146	1.276	.202	.180	Not support
H3	PM→FL	.177	−1.272	.203	−.208	Not Support
H4	CS→FL	.211	3.959	***	.781	Support
H5	FL→CI	.044	20.877	***	.918	Support

*p < 0.05; **p < 0.01; ***p < 0.001.

Of the five hypotheses proposed, three were supported. Hypothesis 1 predicted that perceived cost (PC) had a positive impact on flow, and the results supported this hypothesis. The positive path coefficient (0.224) between the two constructs provided evidence to support H1. Hypothesis 4 predicted that collaboration and share (CS) had a positive impact on flow, and the positive path coefficient between the two constructs (*β* = 0.781, *p* < 0.001) provided evidence to support H4. Hypothesis 5 predicted that flow would have a positive effect on continuance intention (CI) to use collaborative MALL, and the positive path coefficient between the two constructs (*β* = 0.918, *p* < 0.001) indicated that H5 was also supported.

However, contrary to the hypotheses, the results showed that perceived mobility (PM) and social influence (SI) did not have a positive effect on flow, and therefore, H2 and H3 were not supported in the proposed model. Nevertheless, the data analysis revealed that SI had a positive impact, while PM had a negative impact on students’ flow, although neither had statistical significance.

Thus, two factors, PC and CS, were significantly positive influential factors on students’ flow. Therefore, the MCLL Model, including flow and CS, is a better index to predict learners’ intention to use collaborative MALL. The structural equation model (Figure 2) further verifies the position of flow as a key factor in Chinese college students’ intention for further adoption of collaborative MALL. In this new learning approach, CS is the most influential factor on students’ flow, while the other three factors are also influential, but their contributions are less compared to that of CS.

## 5. Discussion

### 5.1. The growing significance of collaborative MALL in language education

The analysis of collaborative MALL experience among college students in China reveals that this approach has become a mainstream learning method in their language learning process. 73% of the surveyed students had collaborative MALL experience, indicating that this approach has been widely adopted. Liberal arts students participated in collaborative MALL activities more frequently, with 16.6% of them taking part in such activities more than three times a week. This demonstrates that collaborative MALL has gradually become an important approach for students to construct and share new knowledge with their peers through interaction, which is a typical characteristic of the information era (Binkley et al., 2012; Ehlers, 2020; Hoi, 2020). This positive message from the survey highlights the importance of collaborative MALL in language education (Liu et al., 2018; Chen et al., 2022; Fu, 2022; Zhang and Zhu, 2022; Dong, 2023).

However, the reported low frequency level and short duration of single collaborative MALL activities suggest that there is still room for improvement in the adoption of this approach among college students in China (Hwang and Fu, 2019; Hsu and Lin, 2021). Furthermore, among those who have no collaborative MALL experience, about 44% were students of liberal arts. As language education in China is more closely related to liberal arts, this result indicates a need for more attention and measures to encourage and enhance the further integration of collaborative MALL in course design and teaching plans (Luo and Chen, 2019; Nguyen and Takashi, 2021). Learner-initiated collaborative MALL activities are more popular among students of science and engineering and students of arts, indicating that language teachers should pay closer attention to technology advances and be more willing to integrate new learning approaches into their teaching practice (Sancho-Gil et al., 2020).

### 5.2. Unveiling the impact of flow on intention for further adoption: hypotheses supported and unsupported

This study proposes a Mobile Collaborative Language Learning Model (MCLL) that integrates antecedents of users’ beliefs and alternative factors to provide a better understanding of the determining mechanism for college students’ intention to learn collaboratively in a mobile environment.

#### 5.2.1. The positive impact of flow on students’ continuance intention: supported hypotheses

Of the five hypotheses proposed, three were supported by the study. The results of the study showed both consistency and inconsistency with the results of previous studies (Taylor and Todd, 1995; Hoffman and Novak, 1996; Hsu and Lu, 2004; Bower et al., 2020; Xu and Zhang, 2020). The findings obtained in this study lead to several insights.

Firstly, the study found that flow significantly impacts college students’ continuance intention to further collaborative MALL experience. The positive effect of flow on the continuance intention of collaborative MALL aligns well with previous studies (Egbert, 2003; Shernoff et al., 2009; Franciosi, 2011; Kiili et al., 2012; Shadiev et al., 2018; Delforge et al., 2019) that have highlighted flow and its positive effect on students in Collaborative MALL as suggested by Nakamura and Csikszentmihalyi (2002). The results indicates the importance of optimizing flow experiences and fostering the state of deep engagement and immersion during the learning process for students.

Secondly, the impact of CS on flow was statistically significant, implying that encouraging collaboration and sharing in the learning process will improve learners’ interest and attention and result in promoted learning engagement. This is also consistent with a number of previous studies varied subjects learning contexts (Dillenbourg, 1999; Leung and Chiu, 2008; Admiraal et al., 2011; Barkley et al., 2014) though related report in MALL is still limited (Ilic, 2015; Al-Ahdal and Alharbi, 2021). Instructors and instructional designers are appealed to consider developing and providing scaffolds and affordances to facilitate students’ collaboration and sharing (Kirsch, 2016; Kukulska-Hulme and Viberg, 2018) to promote better flow in the collaborative MALL experience.

Thirdly, PC is an important factor for students to promote learning flow in collaborative MALL. This may partly due to the dominance of mobile devices in internet access in China. As highlighted by CNNIC (2023), by the end of 2022, an astonishing 99.8% of the 1 trillion internet users accessed the internet *via* their mobile phones. Additionally, Chen et al. (2020) found that 94.48% of college students in their study accessed online learning platforms primarily through their mobile phones, while only 72.88% utilized computers for the same purpose. Researchers (Ogunduyile, 2013; Ilic, 2015) have pointed out that adoption of multiple technologies and media could lead to enhanced collaboration in learning. In the context of collaborative MALL, expenses, time and energy paid by users will promote users’ learning flow. Students’ willingness to pay for knowledge improves, and it is an inevitable trend to respect intellectual property rights and charge for high-quality resources (Head, 2014; Reinhardt and Ross, 2019).

#### 5.2.2. The influence of changing and context-specific learning environments: unsupported hypotheses

The analysis of the data did not support H2 and H3, which aimed to investigate the relationship between perceived mobility (PM), social influence (SI), and flow. These findings deviate from prior research conducted by Venkatesh et al. (2012), Li et al. (2016), and Chen and Wu (2016), which reported consistent results in this regard.

It is possible that the lack of significant effect of PM and SI on Flow in collaborative MALL is due to the fact that these characteristics have become commonplace in the current learning environment, particularly for college students who have grown up with technology and are accustomed to communicating and collaborating online. As such, the perceived mobility and social influences may not have as strong an impact on their learning flow as other factors, such as the challenge and innovation of the learning content, or the degree of collaboration and sharing within the learning process.

However, the non-significant effect of PM and SI on Flow in collaborative MALL may also be context-specific and may not necessarily apply to other groups or contexts. More studies should be conducted to identify the possible causes and lessons that can be learned.

### 5.3. Implications

The findings shed light on several important considerations for designing and implementing effective collaborative Mobile-Assisted Language Learning (MALL) experiences in higher education in China. Specifically, the study highlights the significance of enhancing flow experiences, promoting collaboration and knowledge sharing, and taking into account the cost implications associated with collaborative language learning experiences:

First, to establish an optimal learning environment that cultivates a state of flow, educators can take measures to enhance students’ motivation, concentration, and enjoyment during collaborative language learning activities. Egbert (2003) suggests that learners are more likely to experience flow when engaged in challenging and innovative learning contexts, leading to an increased sense of pleasure and accomplishment. Therefore, language educators and instructors should continuously strive to develop diverse, captivating, and personalized learning materials that cater to the individual needs of students. Additionally, providing prompt and relevant feedback is crucial to encourage active participation and sustained engagement. By incorporating innovative and creative content into collaborative MALL, the meaningfulness of the learning experience can be enhanced, thereby facilitating the emergence of flow experiences among students.

Second, to foster collaboration and knowledge sharing among students, language educators and instructors can implement various strategies. One approach is to establish clear guidelines or rules that encourage active participation and engagement from students in collaborative MALL activities. Assigning tasks that require interactions and cooperation among students can also be beneficial in promoting collaboration. Moreover, language educators and instructors should strive to create a sense of learning community, wherein students feel a sense of belonging and are motivated to exchange ideas and perspectives. This nurturing environment facilitates deeper learning and has the potential to enhance students’ language proficiency and intercultural competence. By emphasizing collaboration and creating a supportive community, language educators can optimize the benefits of collaborative MALL experiences for their students.

Furthermore, it is essential to carefully consider the cost implications of collaborative language learning experiences, given the overwhelming preference for mobile devices in internet usage. Consequently, it becomes crucial to evaluate the financial and time commitments required from students’ perspectives, taking their concerns seriously. Measures should be implemented to ensure that the benefits of collaborative Mobile-Assisted Language Learning (MALL) outweigh the associated costs. By addressing cost-related concerns, educators can foster greater acceptance and adoption of collaborative MALL approaches among college students, leveraging the popularity and accessibility of mobile devices for language learning purposes.

Collaborative Mobile-Assisted Language Learning (MALL) is continuously evolving and gaining popularity as an effective method for language acquisition. By incorporating the suggestions mentioned above, educators have the opportunity to design and implement collaborative MALL experiences that are not only more meaningful but also engaging. As a result, these experiences have the potential to significantly improve language learning outcomes in higher education contexts in China and beyond.

## 6. Conclusion

The current study makes a substantial contribution to the literature on collaborative Mobile-Assisted Language Learning (MALL) by providing valuable insights into the factors influencing college students’ intention to further adopt this approach. The findings emphasize the significant role of flow as a determinant of students’ willingness to engage in collaborative MALL experiences. Additionally, the study highlights the importance of Collaboration and Share (CS) as a key factor that promotes learning flow within the context of collaborative MALL. These findings suggest that language educators and instructional designers should focus on developing innovative and interactive learning content that encourages active participation and fosters collaboration among students in MALL settings.

However, the study also reveals two noteworthy considerations. Firstly, the findings indicate that social influence may not have a significant impact on predicting the adoption of new learning approaches, challenging the commonly assumed influence of social factors. Secondly, the study suggests that perceived mobility may not be as influential as previously believed in shaping students’ intentions toward collaborative MALL. These findings call for further investigation and a re-evaluation of the relationships between these factors in different educational contexts and with diverse groups of learners.

It is important to acknowledge certain limitations of the study. Firstly, this research is exploratory in nature, primarily focusing on the relationship between the identified factors and flow within collaborative MALL experiences. Consequently, only prominent factors were considered, and the data primarily comprised responses from college students in China. Secondly, the study did not collect data on participants’ learning outcomes before and after adopting the collaborative learning approach, which restricts the assessment of changes in their overall learning performance.

To advance the field, future research should delve into investigating the factors that influence students’ intention to further adopt the Mobile Collaborative Language Learning (MCLL) model. This should include an examination of the relationships between perceived mobility, social influences, and flow in diverse settings or with different cohorts of learners, aiming to develop a more comprehensive understanding of their impact on MCLL.

Additionally, further studies are warranted to validate the proposed model, refine the role of flow, and explore the influence of Collaboration and Share (CS) by integrating potential new factors. It is also essential to explore various methodologies for enhancing collaboration through mobile devices and to evaluate the learning outcomes resulting from such approaches in diverse educational settings. Furthermore, given the ongoing advancements in mobile technology, future research should investigate how these technological developments can facilitate and enhance learning outcomes in the context of collaborative MALL.

In conclusion, this study offers valuable insights into the factors influencing college students’ intention to further adopt collaborative MALL. The findings underscore the importance of flow and Collaboration and Share (CS) in driving student engagement and call for further research to refine the model, explore new factors, and examine the impact of collaborative MALL across various educational contexts. These endeavors will contribute to the continued advancement and optimization of collaborative MALL practices, ultimately improving language learning experiences and outcomes for college students.

## Data availability statement

The original contributions presented in the study are included in the article/Supplementary material, further inquiries can be directed to the corresponding author.

## Ethics statement

Ethical review and approval was not required for the study on human participants in accordance with the local legislation and institutional requirements. The patients/participants provided their written informed consent to participate in this study.

## Author contributions

LH: conceptualization, project administration, writing—review and editing, and supervision. DH: validation, data curation, formal analysis, and writing—original draft. HW and XD: research instrument design, data collection, and sorting. All authors contributed to the article and approved the submitted version.

## Conflict of interest

The authors declare that the research was conducted in the absence of any commercial or financial relationships that could be construed as a potential conflict of interest.

## Publisher’s note

All claims expressed in this article are solely those of the authors and do not necessarily represent those of their affiliated organizations, or those of the publisher, the editors and the reviewers. Any product that may be evaluated in this article, or claim that may be made by its manufacturer, is not guaranteed or endorsed by the publisher.
