# Supplementary material for: Chinese college students collaborative mobile-assisted language learning experience and flow as a key factor for further adoption
Source: Front Psychol. 2023 Sep 13;14:1165332. doi: 10.3389/fpsyg.2023.1165332 (PMC10525693; doi:10.3389/fpsyg.2023.1165332)
Supplement: Supplementary file 1 [file Table_1.DOCX]

**The Questionnaire of College Students'
Collaborative MALL Experience in China**

Welcome to our survey about your experience of collaboration in Mobile assisted language learning and some factors that have influenced your experience. The survey will take about 10 minutes to complete. The survey is for academic purpose only, and data collected will be applied to academic analysis exclusively. Your anonymity will be guaranteed. There is no right or wrong answer for each item of the survey.

We sincerely appreciate your support! !

**ⅠBasic personal information**

1.Gender

A. Male B. Female

2. Age range

A. 18-22 years old B. 23-27 years old C. 28-31 years old D. More than 32 years old

3. Educational background

A. Vocational school students B. Undergraduate students C. Graduate students

4. Major

A. Science and engineering B. Liberal arts C. Arts

**Ⅱ Premise measurement questions**

1. Did you have any experience of Collaborative MALL* ?

A. Yes B. No

Note: Collaborative MALL refers to the act of learning a foreign language through online discussions or other forms of collaboration using mobile devices such as mobile apps or tablets.

**Ⅲ Collaborative MALL experience**

2. How long have you experienced Collaborative MALL ?

A. < 3 months

B. >3-6 months

C. >1-2 years

D. >2 years

3. How often did you take part in Collaborative MALL in the last 3 months?

A. 1-2 times every 3 months

B. 1-2 times every 2 months

C. 1-2 times per month

D. 1-2 times per week

E. More than 3 times per week

4. How long was your Collaborative MALL activity each time in average?

A. < 30 minutes

B. > 30 minutes-1 hour

C. > 1 hour-2 hours

D. > 2 hours

5. What are the two most commonly adopted Collaborative MALL activities in the past three months? （2 options can be selected）

A. Discussion and exploration grouping（Learners use mobile media technology workshops to collaborate on presentations, reports, essays, research reports, etc.）

B. Course required grouping task（Learners help each other to complete the course tasks in the online course）

C. Always with you collaboration（Learners work in teams at odd hours to learn and collaborate）

D. Online self-study grouping（Learners access online study rooms together to monitor and motivate each other）

**Ⅳ Influential factors scale**

**(**This section is based on your most recent Collaborative MALL experience)

1. Perceived ease of use

(1) My Mobile Collaborative language learning device is portable

Completely disagree 1 2 3 4 5 Completely agree

(2) My Mobile Cooperative language learning materials is easy to carry and access

Completely disagree 1 2 3 4 5 Completely agree

2. Collaboration and sharing

(1) The learning resources shared in Collaborative MALL fulfil my needs

Completely disagree 1 2 3 4 5 Completely agree

(2) In Collaborative MALL, I successfully complete collaboration with team members

Completely disagree 1 2 3 4 5 Completely agree

(3) Collaboration with team members in Collaborative MALL helps mecomplete the learning task successfully

Completely disagree 1 2 3 4 5 Completely agree

3. Social influence

(1) I participate in Collaborative MALL because my teacher recommend

Completely disagree 1 2 3 4 5 Completely agree

(2) I participate Collaborative MALL because my classmates and friends recommend

Completely disagree 1 2 3 4 5 Completely agree

(3) I participate Collaborative MALL because medias (advertising, mobile app) recommend so

Completely disagree 1 2 3 4 5 Completely agree

4. Flow

(1) I am immersed in learning content in Collaborative MALL

Completely disagree 1 2 3 4 5 Completely agree

(2) In Collaborative MALL, I am happy and feel that time passes quickly

Completely disagree 1 2 3 4 5 Completely agree

(3) Collaborative MALL enables me to learn for a long time

Completely disagree 1 2 3 4 5 Completely agree

5. Perceived cost

(1) I think the price of information services paid for Collaborative MALL is high.

Completely disagree 1 2 3 4 5 Completely agree

1. I think Collaborative MALL consumes high cellular data flow

Completely disagree 1 2 3 4 5 Completely agree

6. Performance expectations

(1) Collaborative MALL can improve my learning efficiency

Completely disagree 1 2 3 4 5 Completely agree

(2) Collaborative MALL enables me to learn more knowledge

Completely disagree 1 2 3 4 5 Completely agree

(3) Collaborative MALL can improve my learning effect

Completely disagree 1 2 3 4 5 Completely agree

7. Continuance intention

(1) I am still using Collaborative MALL

Completely disagree 1 2 3 4 5 Completely agree

(2) I will continuously use Collaborative MALL in the future

Completely disagree 1 2 3 4 5 Completely agree

(3) I am willing to recommend Collaborative MALL to others

Completely disagree 1 2 3 4 5 Completely agree

**That is end of the survey! Thank your very much for your effort!**
